# Adipose-Derived Stem Cell Therapy Attenuates HIF-1α/mTOR/REDD1 Signaling in Obese Hypertensive Rats

**DOI:** 10.1186/s43556-025-00288-1

**Published:** 2025-10-03

**Authors:** Renata Nakamichi, Mario Luis Ribeiro Cesaretti, Eric Rafael Andrade Silva, Camila Nunes Oliveira, Evelyn Manuella Martins Gomes Jodas, Miguel Cendoroglo Neto, Beata Marie Redublo Quinto, Marcelo Costa Batista

**Affiliations:** 1 Nephrology Division, Department of Medicine, Universidade Federal de São Paulo, Pedro de Toledo 669, 10 Andar, Vila Clementino, zip code: , 04039-032 Brazil; 2 Hospital Israelita Albert Einstein, São Paulo, Brazil

## Dear Editor,

Visceral obesity is a key component of metabolic syndrome, a condition strongly associated with an increased risk of kidney and cardiovascular diseases (CVD) [1]. Fat accumulation in the abdominal region induces inflammation, elevating pro-inflammatory markers such as TNF-α and leptin, while reducing anti-inflammatory adipokines like adiponectin [2]. Hypoxia plays a critical role in this metabolic dysfunction through the upregulation of HIF-1α, a key regulator of cellular responses to low oxygen levels [3]. The increased expression of HIF-1α in kidney disease suggests its involvement in glomerular injury and renal dysfunction [4]. Recent studies have highlighted the therapeutic potential of adipose tissue-derived stem cells (ASCs) for obesity-related complications. ASCs secrete bioactive molecules that modulate inflammation, support tissue repair, and improve metabolic profiles [5]. This study aimed to evaluate the effects of ASC therapy on kidney disease progression and metabolic dysfunction in spontaneously hypertensive rats (SHRs) fed a high-fat diet, with a particular focus on the HIF-1α/mTOR/REDD1 signaling pathway.

Throughout the study, no significant changes in blood pressure or body weight were observed in any group. ASC administration did not affect tail arterial pressure, and body weight remained stable across all conditions. The high-fat diet, however, significantly increased visceral fat compared to controls, an effect reversed by ASC treatment, which markedly reduced visceral fat in this group. No significant changes were noted in control animals or in those treated with ASC1 or ASC2. Insulin resistance, confirmed by reduced glucose uptake in the high-fat diet group, was significantly improved by ASC therapy, which enhanced glucose uptake by 75.89%, indicating a reversal of insulin resistance and improved metabolic function.

Dyslipidemia, characterized by elevated triglycerides (27%) and LDL (16%) and reduced HDL (36.4%), was induced by the high-fat diet. ASC treatment for two weeks (ASC2) significantly improved the lipid profile, reducing triglyceride and LDL levels by 44% and 29%, respectively, and increasing HDL by 44% compared to untreated high-fat diet rats, suggesting a restorative effect on lipid balance in visceral obesity. In parallel, the high-fat diet also induced kidney injury, as evidenced by elevated renal biomarkers including NGAL (44%), KIM1 (2%), and cystatin C (28%). ASC2 treatment significantly improved renal function by reducing NGAL by 98%, KIM1 by 4%, cystatin C by 44%, and proteinuria by 61%, supporting a protective role of ASC therapy against obesity-induced kidney damage.

ASC treatment also reversed alterations in adipokine levels caused by the high-fat diet. The diet significantly reduced adiponectin levels by 68% and increased leptin levels in epididymal tissue by 88%. ASC therapy restored adiponectin levels and reduced leptin production by 87% and 64%, respectively, suggesting a restoration of adipokine balance. Furthermore, ASC treatment reduced TNF-α production in both epididymal and renal tissues. In high-fat diet rats, TNF-α levels and gene expression were significantly elevated. ASC therapy reduced TNF-α protein levels by 42% and gene expression by 93%, highlighting its anti-inflammatory effects.

**Fig. 1:**
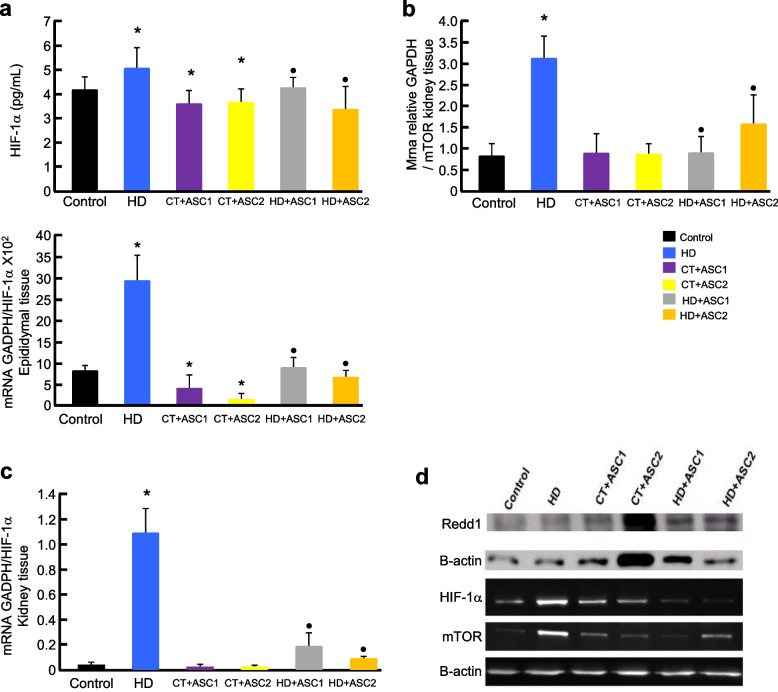
Effects of adipose-derived stem cell (ASC) treatment on visceral fat, HIF-1α expression, and the mTOR/REDD1 pathway in spontaneously hypertensive rats (SHRs) with high-fat diet-induced obesity. (a) Plasma concentration of HIF-1α (top) and mRNA expression of HIF-1α in epididymal adipose tissue (bottom). (b) tissue mTOR mRNA expression in kidney tissue. (c) HIF-1α mRNA expression in kidney. (d) Western blot analysis of REDD1, HIF-1α, and mTOR protein expression in kidney tissue. ASC treatment significantly reduced both gene and protein expression of HIF-1α, REDD1, and mTOR in animals fed a high-fat diet, suggesting attenuation of hypoxia and inflammatory pathways. Data are presented as mean ± SEM (n = 6 per group). *p < 0.05 vs. Control; •p < 0.05 vs. HD. Bar color legend: Black = Control; Blue = HD (high-fat diet); Purple = CT + ASC1; Yellow = CT + ASC2; Gray = HD + ASC1; Orange = HD + ASC2

HIF-1α expression in visceral adipose tissue was significantly increased in the high-fat diet group. ASC treatment significantly reduced HIF-1α levels, indicating reduced tissue hypoxia. These results are shown in Fig. 1 (a), which presents plasma concentrations and gene expression of HIF-1α in epididymal tissue.

In kidney tissue, high-fat diet rats exhibited increased gene and protein expression of HIF-1α, REDD1, and mTOR. ASC treatment significantly decreased the expression of these markers, suggesting reduced hypoxic and inflammatory signaling. These effects are illustrated in Fig. 1, (b–d), showing changes in gene expression of HIF-1α (b), mTOR (c), and REDD1 (d). The expression of mTOR and HIF-1α correlated positively with metabolic disturbances, kidney disease progression, lipid abnormalities, and inflammation. Plasma HIF-1α was positively correlated with leptin (r = 0.824, p < 0.001), kidney HIF-1α (r = 0.901, p < 0.001), mTOR (r = 0.431, p < 0.007), HDL (r = 0.674, p < 0.001), triglycerides (r = 0.362, p < 0.026), proteinuria (r = 0.767, p < 0.001), and KIM1 (r = 0.889, p < 0.001), and negatively correlated with adiponectin (r = −0.667, p < 0.004). mTOR expression also positively correlated with REDD1 (r = 0.516, p < 0.001), creatinine (r = 0.363, p < 0.010), TNF-α (r = 0.617, p < 0.001), KIM1 (r = 0.414, p < 0.010), and cystatin C (r = 0.335, p < 0.040), and negatively correlated with HDL (r = −0.515, p < 0.002) and creatinine clearance.

A high-fat diet in SHRs led to significant adipose tissue expansion and impaired glucose metabolism, consistent with insulin resistance and glucose intolerance. Elevated circulating free fatty acids contributed to dyslipidemia, insulin resistance, and other metabolic disturbances commonly seen in obesity [1]. Additionally, the diet induced local inflammation, evidenced by increased TNF-α and leptin levels and reduced adiponectin, which worsened renal function [2]. Hypoxia, mediated by HIF-1α, emerged as a central factor in metabolic and renal dysfunction. The upregulation of HIF-1α in both adipose and renal tissues was associated with increased inflammation, supporting the interplay between obesity, hypoxia, and inflammatory damage [3]. Furthermore, elevated mTOR expression in the kidneys—commonly observed in obesity and insulin resistance—suggests its contribution to kidney injury [4]. ASC therapy improved insulin sensitivity, glucose homeostasis, lipid profiles, and inflammation, and restored adiponectin levels [5]. It also enhanced renal function, likely through downregulation of the HIF-1α and mTOR pathways, and reduced REDD1 expression, a critical mediator of inflammation and hypoxia [5]. Collectively, these findings suggest that ASC therapy may offer a promising strategy to mitigate obesity-induced metabolic and renal dysfunction.

Although this study assessed HIF-1α expression in both adipose tissue and kidneys, it did not evaluate lipid accumulation specifically in kidney tissue. Given its potential contribution to renal dysfunction, future studies should include targeted interventions in the mTOR/HIF-1α/REDD1 pathway to establish causal relationships and move beyond correlational analysis. Further investigation is also needed to elucidate the paracrine mechanisms by which ASCs modulate these pathways, and to evaluate lipid deposition in the kidneys as a possible contributor to renal impairment.

In conclusion, this study demonstrates that ASC therapy can ameliorate metabolic and renal dysfunction caused by a high-fat diet in SHRs. By reducing inflammation and modulating key signaling pathways—HIF-1α, mTOR, and REDD1—ASC therapy shows promise as a therapeutic approach for obesity-related complications. Further studies are warranted to elucidate the underlying mechanisms of action.

## Supplementary Information

Supplementary Material 1.

## Data Availability

The data supporting the findings of this study—including lipid profiles, kidney function metrics, and adipocytokine levels—are not publicly available. However, they are available from the corresponding author upon reasonable request. Data access will be provided subject to ethical and privacy considerations.
