# Supplementary material for: Adipose-Derived Stem Cell Therapy Attenuates HIF-1α/mTOR/REDD1 Signaling in Obese Hypertensive Rats
Source: Mol Biomed. 2025 Oct 3;6:70. doi: 10.1186/s43556-025-00288-1 (PMC12491122; doi:10.1186/s43556-025-00288-1)
Supplement: Supplementary file 1 — Supplementary Material 1. [file 43556_2025_288_MOESM1_ESM.docx]

**Adipose-Derived Stem Cell Therapy Attenuates HIF-1α/mTOR/REDD1 Signaling in Obese Hypertensive Rats**

Renata Nakamichi^1^, Mario Luis Ribeiro Cesaretti^1^, Eric Rafael Andrade Silva^1^, Camila Nunes Oliveira^1^, Evelyn Manuella Martins Gomes Jodas^1^, Miguel Cendoroglo Neto^2^, Beata Marie Redublo Quinto^1*^, Marcelo Costa Batista^1,2^

1. Nephrology Division, Department of Medicine, Universidade Federal de São Paulo, Brazil

2. Hospital Israelita Albert Einstein, São Paulo, Brazil

*Corresponding author: Beata Marie Redublo Quinto

**Experimental Procedure**

This section contains detailed methods and protocols that complement the main content of the study. The following supplementary materials include specific procedures used in the experiment, which are essential for replicating the results and understanding the experimental setup.

**Animals:** This study used 12-week-old male SHR rats obtained from UNIFESP. Animals were housed in standard conditions with free access to food and water. Rats with baseline blood pressure over 165 mmHg were selected for the study. The rats were divided into different groups: control SHR, high-fat diet (HD), and groups treated with adipose tissue-derived stem cells (ASC) after receiving a high-fat diet (HD+ASC1, HD+ASC2). The high-fat diet (3:2:2:1 ratio of standard chow, roasted peanuts, milk chocolate, and corn starch cookies) was administered for 12 weeks.

**Isolation of ASC:** Male SHR rats on standard chow were euthanized by an overdose of peritoneal anesthetic (2mg/kg xylazine, 360mg/kg ketamine). Following euthanasia, visceral adipose tissue was collected from the abdominal region, washed with PBS, and digested with 0.075% collagenase IA in DMEM with 1% glucose. After filtering and centrifuging the tissue, the cell pellet was suspended in 4% NH4Cl to remove red blood cells, then washed and plated in DMEM with 10% FBS. Cells were cultured until approximately 80% confluence and then subcultured through the fifth passage. Potential confounders, such as diet composition, genetic variability, and environmental conditions, were carefully controlled to ensure the effects observed were mainly due to the intervention.

**Validation and Differentiation of Adipose Tissue-Derived Stem Cells:**
The ASCs were analysed by determining the characteristics of their cell surface by flow cytometry using the FACSanto cytometer (Becton Dickinson, NJ, USA) (Figure 1).

Cells were differentiated into adipocytes and osteocytes using adipogenic culture medium (StemPro Adipogenesis Differentiation Kit, Thermo Fisher, MA, USA) and osteogenic medium (StemPro Osteogenesis Differentiation Kit, Thermo Fisher), respectively, according to the manufacturer’s recommendations

**Oral Glucose Tolerance Test (OGTT):** Rats were fasted for 12 hours and then administered 68 mg/kg glucose. Blood glucose levels were measured at various time points (0, 15, 30, 60, 90, and 120 minutes) to assess glucose metabolism. The area under the glucose curve (AUC) was calculated using GraphPad Prism.

**Insulin Tolerance Test (ITT):** Two days post-OGTT, insulin sensitivity was assessed by administering 2 mU/kg insulin intraperitoneally after a 6-hour fast. Blood glucose levels were measured at multiple time points (0, 5, 10, 15, 20, 25, and 30 minutes). The AUC for blood glucose was calculated.

**Lipid Profile:** After euthanasia, plasma lipid levels (cholesterol, LDL, HDL) were measured using colorimetric methods. The visceral fat index was calculated by dividing the total fat weight by body weight.

**Renal Functional Parameters:** 24-hour urine was collected to assess renal function. Urinary volume, protein, and creatinine concentrations were measured. Neutrophilic gelatinase-associated lipocalin (NGAL) and serum cystatin C levels were quantified using ELISA. Creatinine clearance was calculated using plasma creatinine measurements.

**Adipokine and Cytokine Analysis:**Plasma levels of adiponectin, leptin, TNF-α, and HIF-1α were measured by ELISA.

**mRNA Extraction and cDNA Synthesis:** RNA was extracted from renal and epididymal tissues using the QuantiTect kit and reverse transcribed into cDNA for further analysis.

**Gene Expression Analysis by Real-Time PCR:** Reactions were performed using specific primers and SYBR Green dye. The expression levels of target genes were normalized to GAPDH. The primers for the genes of interest were designed using the Primer Express® program (Applied Biosystems, CA, USA) and based on the sequence of genes obtained from GenBank: Epididymal fat: adiponectin (sense:GGTGACCAGGAGATGCT; anti-sense: TAC-GCTGAATGCTGAGTGATA); leptin (sense: ATGTGGTACGGAAGGTGGAG; anti-sense: TGGCTACCTTCGTCTGTGTG); TNF-α (sense: ACCACAGTCCATGCCATCAC; an-ti-sense: TCCACCACCCTGTTGCTGTA); HIF-1α: GAACAAAACACAGCGAAGCT; Antisense:TGCAGTGCAATACCTTCCATGT);GAPDH(sense:ACCACAGTCCATGCCATCAC; anti-sense: TCCACCACCCTGTTGCTGTA). Kidney tissue: Adiponectin (sense:GGTGACCAGGAGATGCT; anti-sense:TAC-GCTGAATGCTGAGTGATA); leptin (sense:ATGTGGTACGGAAGGTGGAG; anti-sense: TGGCTACCTTCGTCTGTGTG); TNF-α (sense: ACCACAGTCCATGCCATCAC; an-ti-sense:TCCACCACCCTGTTGCTGTA); HIF-1α(sense:TGCAGTGCAATACCTTCCAT;anti-sense:GAACAAAACACACAGCGAAG); mTOR(sense:TTGCCAACTACCTTCGGAACC;antisense:TCACGGAGAACGAGGACAGC)

**Protein Analysis by Western Blot:** otal protein was extracted from kidney tissue, concentrated using centrifugal filter devices (Millipore, MA, USA), and quantified with the Bradford Protein Assay (Thermo Scientific). 20 µg of protein were separated by SDS-PAGE and transferred to nitrocellulose membranes. The membranes were blocked and incubated with primary antibodies: anti-HIF-1α (monoclonal mouse, Sigma Aldrich), anti-mTOR (polyclonal rabbit, Sigma Aldrich), and anti-β actin (monoclonal mouse, Sigma Aldrich). Following incubation with peroxidase-conjugated secondary antibodies (anti-rabbit and anti-mouse, Millipore), specific proteins were detected using luminol substrate (Immobilon Western Chemiluminescent HRP, Millipore) and analyzed with a Luminescent Image Alliance 4.7 Analyzer (Fuji Photo Film CO, Japan). Protein molecular weights were determined by comparison with standard protein markers (Millipore).

**Statistical Analysis:** Statistical analyses were performed using SPSS 21.0. Data were presented as mean ± standard deviation, and significance was determined using ANOVA with Bonferroni post-hoc test. Correlation analyses were conducted as well. Statistical significance was set at p < 0.05. A power analysis confirmed that a sample size of 6 animals per group was adequate.

**Sample Size Calculation and Ethical Considerations:** Our laboratory adheres to the 3Rs principle (Reduction, Refinement, and Replacement) in animal experimentation. The Reduction principle, which focuses on using the minimum number of animals necessary, is central to our approach. We aim to reduce the number of animals without compromising scientific validity. To achieve this, we base our experiments on previous studies conducted in our lab, where similar numbers of animals were employed. Additionally, we conduct statistical power analyses and calculate effect sizes to ensure that the sample size is adequate for detecting meaningful effects, while minimizing the use of animals. This approach aligns with the 3Rs, particularly the Reduction principle, by ensuring that the number of animals used is optimized for reliable and reproducible results

**Blinding and Allocation:** Group allocation was performed by the bioterist, who was blinded to treatment assignments. Outcome assessments and data analysis were conducted by blinded investigators to ensure objectivity.

**Outcome Measures**

**Metabolic Parameters:** OGTT and ITT were used to assess glucose metabolism and insulin sensitivity, respectively. AUC for glucose and insulin curves was calculated.

**Lipid Profile:** Plasma cholesterol (total, LDL, HDL) levels were measured to assess lipid metabolism.

- **Renal Function:** Creatinine clearance and urinary protein levels were measured to evaluate kidney function. Biomarkers for renal injury (NGAL and cystatin C) were also assessed.
- **Adipose Tissue Analysis:** Visceral fat index was calculated based on fat weight and body weight.
- **Adipokine and Cytokine Levels:** Plasma concentrations of adiponectin, leptin, TNF-α, and HIF-1α were quantified using ELISA.
- **Gene and Protein Expression:** Gene expression of adipokines and mTOR, HIF-1α, and REDD1 was assessed using PCR. Protein levels of these markers were analyzed by Western blot.
